# Anti-Inflammatory Activity of Chitosan and 5-Amino Salicylic Acid Combinations in Experimental Colitis

**DOI:** 10.3390/pharmaceutics12111038

**Published:** 2020-10-29

**Authors:** Henusha D. Jhundoo, Tobias Siefen, Alfred Liang, Christoph Schmidt, John Lokhnauth, Arnaud Béduneau, Yann Pellequer, Crilles Casper Larsen, Alf Lamprecht

**Affiliations:** 1 Department of Pharmaceutics, Institute of Pharmacy, University of Bonn, 53121 Bonn, Germany; hjhundoo@uni-bonn.de (H.D.J.); tobias.siefen@uni-bonn.de (T.S.); 2 Ferring Pharmaceuticals Inc., Parsippany, NJ 07054, USA; Alfred.Liang@ferring.com (A.L.); CrillesCasper.Larsen@ferring.com (C.C.L.); 3 Bayer Consumer Care AG, 4052 Basel, Switzerland; christoph.schmidt@bayer.com; 4 Vytaderm, Fair Lawn, NJ 07410, USA; JohnL@vytaderm.com; 5 PEPITE (EA4267), University of Bourgogne/Franche-Comté, 25000 Besançon, France; arnaud.beduneau@univ-fcomte.fr (A.B.); yann.pellequer@univ-fcomte.fr (Y.P.)

**Keywords:** chitosan, IBD, colitis, inflammation, RAW 264.7 macrophages, 5-amino salicylic acid

## Abstract

Chitosan is used in various drug delivery approaches as a pharmaceutical excipient. Although its potential as an immunomodulatory agent has been reported, its use in this capacity has not been fully explored. The efficacy of chitosan as an active pharmacological agent, particularly in anti-inflammatory therapy in inflammatory bowel diseases (IBD), was investigated in this study. The potential impact of the molecular weight (MW) and degree of deacetylation (DD) of chitosan was investigated together with 5-amino salicylic acid (5-ASA) for its efficacy in a combination anti-inflammatory therapy in murine experimental colitis. Such a combination would potentially be developed into novel dual strategies whereby chitosan acts as a mucoadhesive excipient as well as provide an additional anti-inflammatory benefit. Chitosan grades with different MW and DD were administered intrarectally alone or in combination with 5-ASA to colitis mice for 3 days. Myeloperoxidase (MPO) and alkaline phosphatase (ALP) activity and tumor necrosis factor-α (TNF-α), interleukin-6 (IL-6), interleukin-1β (IL-1β) and nuclear factor kappa-B (NF-κB) levels were assessed from the colon. Intrarectal treatment of colitis with 30 mg/kg chitosan alone and with 30 mg/kg 5-ASA for 3 days led to a significant decrease in MPO, ALP, TNF-α, IL-6, IL-1β and NF-κB in colitis mice compared to untreated mice. Surprisingly, the efficacy of chitosan as an anti-inflammatory polymer was relatively independent from its structural properties, namely DD and MW. However, combinations of chitosan with 5-ASA showed a significant pharmacological improvement, whereby the additive anti-inflammatory efficacy observed shows the possibility of finetuning chitosan by combining it with anti-inflammatory agents to optimize its anti-inflammatory potential.

## 1. Introduction

Inflammatory bowel disease (IBD) is characterized by recurrent episodes of inflammation in the gastrointestinal tract (GIT) with periods of remission and symptoms such as abdominal pain, diarrhoea, rectal bleeding and weight loss. In the treatment of IBD, which is classified into two distinct idiopathic inflammatory diseases, namely ulcerative colitis and Crohn’s disease, anti-inflammatory therapy tends to target known inflammatory mechanisms since the etiology of the disease remains unclear [1,2]. Conventional IBD therapy involves the use of drugs such as anti-inflammatory and immunosuppressant agents such as aminosalicylates (5-amino salicylic acid, 5-ASA), cyclosporine, corticosteroids, thiopurines and sulfasalazine and newer therapies such as anti-TNF-α monoclonal antibodies. However, a high incidence of adverse effects is observed in patients treated with conventional IBD immune suppressive drugs and, besides, antibody-based therapy is expensive and is reserved for later lines when patients show no response to drugs such as salicylates [3,4]. Although 5-ASA has been developed using various formulation strategies that enhance its delivery to the rectal or colonic site of inflammation in colitis, e.g., rectal enemas, foam gels or as controlled release preparations or as prodrugs, anti-inflammatory therapy in IBD still remains a challenge and has to date not been optimized for every patient. The incidence of treatment-emergent adverse events with 5-ASA therapy in IBD shows that alternative therapies are required for certain patients [5,6,7]. While most approaches focus on the design of delivery systems that specifically release the drug to the site of inflamed tissue by selecting excipients to optimize the delivery, the potential anti-inflammatory effect of excipients as single agents or in combination with other drugs has been hardly analyzed to date. This could be of major benefit in therapy since the known active pharmaceutical ingredient could be complemented by an excipient that not only fulfils formulation-related functions but also provides a direct therapeutic effect itself. One of the first examples for such therapeutic strategy is the use of chitosan. Interestingly, the anti-inflammatory effects of chitosan and its oligosaccharides have been reported in numerous studies [8,9,10,11,12,13,14,15,16,17,18,19,20,21,22,23,24]. Additionally, chitosan has been found to exhibit noteworthy biological effects, namely anti-microbial, hypocholesterolemic, immune-boosting and anti-tumor properties [25,26]. It has been suggested that the biological activities of chitosan may depend on the molecular weight (MW) and the degree of deacetylation (DD) of the parent chitosan material and these properties are inherently affected by the distribution pattern of d- glucosamine (GlcN) and β-1,4-linked *N*-acetyl-d-glucosamine (GlcNAc) along the oligomeric chain of the parent chitosan [26,27,28,29].

The anti-inflammatory effects of chitosan (ChS) and its oligosaccharides were reported a few years ago [8,9,10]. Although chitosan-based pellets showed enhanced efficacy in the treatment of experimental colitis [11] and that chitosan has a protective effect against intestinal inflammation [8], a detailed characterization of the polymer properties such as molecular weight (MW) and the degree of deacetylation (DD) and their potential influence on the therapeutic effect is missing. In order to investigate the relationship between structure and anti-inflammatory properties of chitosan in a systemic way, defined fractions of chitosan with different MW and DD were studied for their therapeutic benefit in experimental murine colitis.

As mentioned earlier, preliminary findings indicated a certain limitation of anti-inflammatory effects for chitosan alone and therefore the potential therapeutic use of chitosan in IBD would be surely preferable in combination with a well-established conventional drug. Accordingly, the focus of this study was therefore to elucidate the pharmacological anti-inflammatory effect of chitosan and chitosan and 5-ASA combinations in a murine experimental colitis model.

## 2. Materials and Methods

### 2.1. Materials

5-Amino salicylic acid (5-ASA) was obtained from Sigma-Aldrich (Deisenhofen, Germany). The chitosan derivatives with varying viscosity and degree of deacetylation were purchased from Heppe Medical Chitosan GmbH (Halle, Germany). The MW, viscosity and DD of each chitosan grade are listed in Table S1 (Supplementary File). All tested chitosan grades were free of endotoxin. Lipopolysaccharides (LPS) derived from *Escherichia coli* 0111:B4 were purchased from Sigma-Aldrich (Taufkirchen, Germany). All other reagents for cell culture were purchased from Biochrom GmbH (Berlin, Germany). All other chemicals used were obtained from Sigma Aldrich (Taufkirchen, Germany).

### 2.2. Animal Treatment

Animal experiments were conducted using male Swiss/CD-1 mice (4–6 weeks old, average weight = 25 g) purchased from Janvier (Saint-Berthevin, France). All animal experiments were performed in accordance with the recommendations in the Guide for the Care and Use of Laboratory Animals (N01-OD-4-2139 Task Order #188, 2011, Institute of Laboratory Animal Resources, National Research Council, National Academy of Sciences, US). Experiments were performed at the University of Burgundy/Franche-Comté in Besançon, France in compliance with French legislation on animal experimentation under experimentation authorization no. A-25-48. The 2,4,6-trinitrobenzenesulfonic acid (TNBS) colitis model was used as it enables colitis induction at an exact predetermined location [12]. All mice (*n* = 5) were acclimatized to laboratory conditions for one week preceding the start of the experiments with food and water ad libitum. Food was withdrawn from the animals 24 h before the start of the experiment, although water was provided. The mice were lightly narcotized using isoflurane prior to intrarectal catheterization (4 cm) to insert 100 µL of TNBS in 50% ethanol at a dose of 90 mg/kg body weight. The mice were kept for 24 h without any treatment to allow the full development of colitis. Subsequently, the mice were treated with 30 mg/kg body weight of chitosan solutions made up using different grades of chitosan. The treatment was also administered using intrarectal catheterization (4 cm) in a volume of 100 µL for three consecutive days to ensure a full compound deposition at the site of inflammation. The control group received saline solution for three consecutive days after induction of colitis with TNBS. The mice were sacrificed 24 h after the last treatment. The colon was resected and washed with 1 mL cold PBS prior to storage in an appropriate buffer solution.

### 2.3. Clinical Activity Score and Colon Weight/Length Index

A clinical activity score (CAS) was used to determine the extent of inflammation in the animals from the assessment of the body weight, stool consistency and presence of rectal bleeding. The percentage of weight loss was obtained from the loss of baseline body weight and allocated a score. The presence of blood in stools and diarrhoea was assessed using a scoring system. The mean of these three parameters formed the clinical activity score which ranged from 0 (healthy) to 6 (maximum colitis) [13]. The colon weight/length index (CWL) was calculated as the ratio of the weight of the inflamed colon to the total length of the colon.

### 2.4. Assessment of the Inflammatory Biomarkers

Distal colonic tissues including the macroscopically visible inflamed gut regions were minced in 1 mL buffer solutions and subjected to homogenization using the Ultra-Turrax^®^ (IKA, Staufen, Germany) at 10,000 rpm for one min and this procedure was conducted for three freeze–thaw cycles. The homogenates were centrifuged at 10,000 rpm at 4 °C for 10 min and the supernatant was collected and used for the analysis of the inflammatory biomarkers. The myeloperoxidase (MPO) activity was determined according to a colorimetric standard method [14]. Levels of alkaline phosphatase (ALP) were measured using the SensoLyte^®^ pNPP Alkaline Phosphatase Assay Kit (Freemont, California, CA, USA) and tissue concentrations of pro-inflammatory cytokines, TNF-α and IL-6 were assessed from the homogenates using commercial ELISA kits (eBioscience, Vienna, Austria).

The total mouse nuclear factor kappa-B (NF-κB) p65 levels in the colonic homogenates were measured using a commercial ELISA kit engineered for a fast analysis of samples, the NF-κB p65 (Total/Phospho) Human InstantOne™ ELISA Kit (eBioscience, Austria). The reagents used in a traditional sandwich ELISA were added in solution to a pre-coated plate followed by a wash step and detection with the TMB (3,3′,5,5′-tetramethylbenzidine) colorimetric substrate. NF-κB p65 is one of the two subunits of NF-κB that heterodimerizes with the other subunits, p50 or p52. The binding of TNF-α to its cognate receptor phosphorylates the inhibitory kappa kinase (IκK) which in turn phosphorylates IκB, allowing proteasomal degradation of IκB. Phosphorylation of p65 at Ser 536 located in the carboxy-terminal transactivation domains (TADs) leads to nuclear localization of the transcriptionally active complex and NF-κB-mediated transactivation of several downstream genes. It specifically plays a key role in transcription of immunoglobulin κ, which is why the detection of total NF-κB p65 vs. phosphorylated NF-κB p65 was used for the evaluation of NF-κB activity and as an indirect measure of TNF-α binding.

### 2.5. Bioadhesion Studies

Chitosan was labeled using fluorescein isothiocyanate (FITC) as described previously [15]. FITC-labeled chitosan was freeze dried and the FITC labeling efficiency was determined by measuring the fluorescence emission of FITC–chitosan solution against standard solutions of FITC. The FITC labeling amount was found to be 2.8±1.0 *w/w* % of FITC to FITC–chitosan.

One hundred microliters of the labeled chitosan solution were administered intrarectally and the colon was resected 24 h later and stored at −20 °C. Cryosections of a thickness of 13 µm were prepared using a cryomicrotome (Slee, Mainz, Germany) prior to the visualization of the sample using confocal laser scanning microscopy (CLSM) (Nikon Instruments Europe B.V., Amsterdam, Netherlands). Image analysis was conducted using ImageJ software (NIH, Maryland, MD, USA) (*n* = 3). The corrected total fluorescence (CTF) was calculated as the difference between integrated density and (area of selected cell or tissue × mean fluorescence of background readings). The images obtained from the CLSM were analyzed by setting a threshold using the thresholding tool [16].

### 2.6. Cell Culture Studies

RAW 264.7 mouse macrophage cells were purchased from ATCC, ATCC^®^-TIB-71 (Wesel, Germany) were cultured in endotoxin-free Dulbecco’s modified Eagle’s medium containing 5.5 mmol/L glucose, 2 mmol/L glutamine, 1 mmol/L sodium pyruvate and supplemented with 10% foetal bovine serum, 100 U/mL penicillin and 0.1 mg/mL streptomycin. RAW 264.7 cells were seeded in 96-well plates at a density of 1 × 10^5^ cells/well and allowed to grow for 24 h (day 1). The medium was aspirated prior to the addition of serum-free medium containing LPS (1 µg/mL) to the cells, which were incubated for another 24 h at 37 °C (day 2). On day 3, medium was aspirated and cells were incubated with chitosan solutions at concentrations of 30 and 300 µg/mL made up in serum-free medium alone and in combination with 30 µg/mL 5-ASA. The supernatants from the stimulated cells were collected after 24 h of incubation (day 4) and used for quantification of TNF-α using a commercial ELISA kit (eBioscience, Vienna, Austria).

In vitro NF-κB activity was assessed using RAW-Blue™ cells (RAW-SP) purchased from InvivoGen (Toulouse, France) and derived from murine RAW 264.7 macrophages that contain a chromosomal integration of a secreted embryonic alkaline phosphatase (SEAP) reporter construct, which is driven by nuclear factor kappa-B (NF-κB) and alkaline phosphatase-1 (ALP-1). RAW-Blue™ cells were cultured in DMEM containing l-glutamine, 100 µg/mL normocin and 100 µg/mL zeocin according to the manufacturer’s recommendations. The NF-κB activity was measured from the levels of SEAP released in the cell supernatant using a colorimetric enzymatic assay. RAW-Blue™ cells were grown and incubated with chitosan solutions at different concentrations as described earlier. The supernatants were incubated with Quanti-Blue™ (InvivoGen) 50% (*v/v*) for 24 h at 37 °C and the absorbance was measured at 650 nm (Victor 3V, Perkin Elmer, Waltham, MA, USA).

### 2.7. Cytokine Array for RAW 264.7 Macrophage Cell Line

A Proteome Profiler Cytokine Array Kit (R&D Systems, Minneapolis, USA) was used on RAW 264.7 cells purchased from ATCC (Wesel, Germany) according to the supplier’s instructions. Cells were either untreated or incubated with 1 µg/mL LPS or 10 µg/mL chitosan (chitosan 70-10) or 1 µg/mL LPS + 10 µg/mL chitosan for 24 h and supernatants were diluted and mixed with the biotinylated detection antibodies. The antibody mixtures were thereafter incubated on a nitrocellulose membrane on which carefully selected capture antibodies were pre-spotted in duplicate. After a washing step to remove the unbound material, streptavidin–HRP and chemoluminescent detection reagents were added sequentially prior to the development of the membrane using an X-ray film for 5 min. The pixel densities were analyzed using image analysis software, Image Lab 3.0.1 (Beta 2) (Biorad Laboratories, Hercules, California, USA), and the corresponding signals on different arrays were compared to determine the relative change in cytokine levels between samples.

### 2.8. Statistical Analysis

Statistical analysis was conducted using Sigmastat 4.0 software (Systat Software, Inc., San Jose, California, USA). Statistical difference was determined using Kruskal–Wallis ANOVA and followed by multiple comparisons using Dunnett’s test. The data were expressed as mean ± SD and treatments were considered significantly different if *p* < 0.05.

## 3. Results

### 3.1. Anti-Inflammatory Effects In Vitro

**Figure 1:**
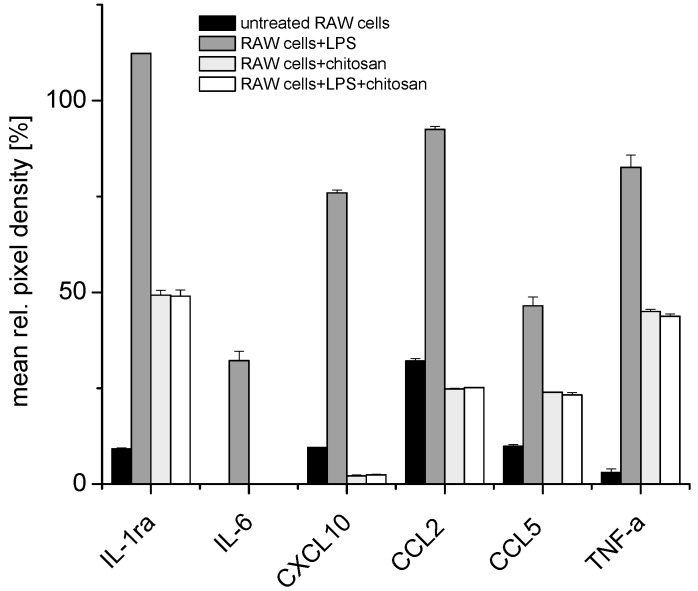
Cytokine array for mouse RAW 264.7 macrophage cells that were either untreated, treated with 1 µg/mL lipopolysaccharide (LPS) for 24 h, treated with 10 μg/mL chitosan for 24 h or treated with 1 µg/mL LPS for 24 h and with 10 μg/mL chitosan for another 24 h (for full data set, see Supplementary Figure S1).

A cytokine array revealed increasing levels of pro-inflammatory cytokines, such as TNF-α, IL-1β and IL-6 and chemokines such as CCL5 (RANTES), CXCL2 (MIP-2α) and CCL2 (MCP-1) in RAW 264.7 cells after stimulation with LPS for 24 h, while, conversely, the treatment with 10 µg/mL chitosan with or without prior LPS led to a reduction in the levels of pro-inflammatory cytokines, such as TNF-α, IL-1β and IL-6 and chemokines such as CCL5 (RANTES) or CCL2 (MCP-1), after stimulation with 1 µg/mL of LPS for 24 h. Figure 1 shows that IL-1ra, IL-6 and TNF-α are only expressed after LPS stimulation in RAW 264.7 macrophages. IL-10, which was also expressed after LPS stimulation, is known to exert an anti-inflammatory effect to restore the exaggerated immune response back to its normal state. These studies revealed that it would be logical to investigate the concentration of TNF-α, IL-6 and IL-1β as representative pro-inflammatory cytokines produced by RAW 264.7 cells during further studies. In addition, the production of granulocyte–macrophage colony-stimulating factor (GM-CSF) was reduced in the presence of chitosan.

**Figure 2:**
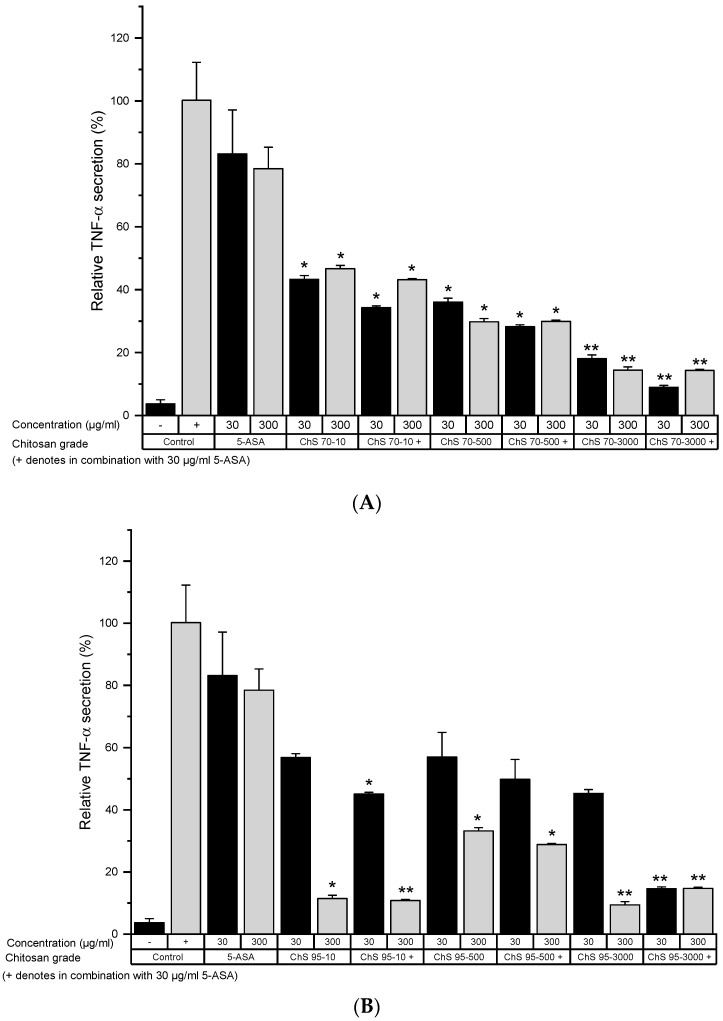
Tumor necrosis factor-α (TNF-α) secretion from LPS-stimulated RAW 264.7 cells after incubation with 30 and 300 µg/mL of chitosan 70% (**A**) and 95% (**B**) degree of deacetylation (DD) and viscosity 10, 500 and 3000 mPas for 24 h in serum-free medium (−): healthy control, (+): untreated colitis control (mean ± SD; *n* = 6; * *p* < 0.05; ** *p* < 0.01 compared to the 5-amino salicylic acid (5-ASA) 30 µg/mL control, Mann–Whitney test).

For more specific results, TNF-α and NF-κB levels were recorded in LPS-activated cells after treatment with different chitosan grades. Treating cells with 30 μg/mL 5-ASA alone led only to a slight reduction in TNF-α levels (Figure 2). In general, the combination of 5-ASA with chitosan led to lower TNF-α levels than chitosan or 5-ASA alone (Figure 1, Supplementary Figure S1), although this effect was insignificant in most groups (Figure 2A–C). With increased viscosity, chitosan reduced TNF-α levels significantly, with grades with 70% DD, while the trend was less clear with 95% DD. A chitosan dose-dependent response was observed with chitosan 95% DD while not with 70% DD.

**Figure 3:**
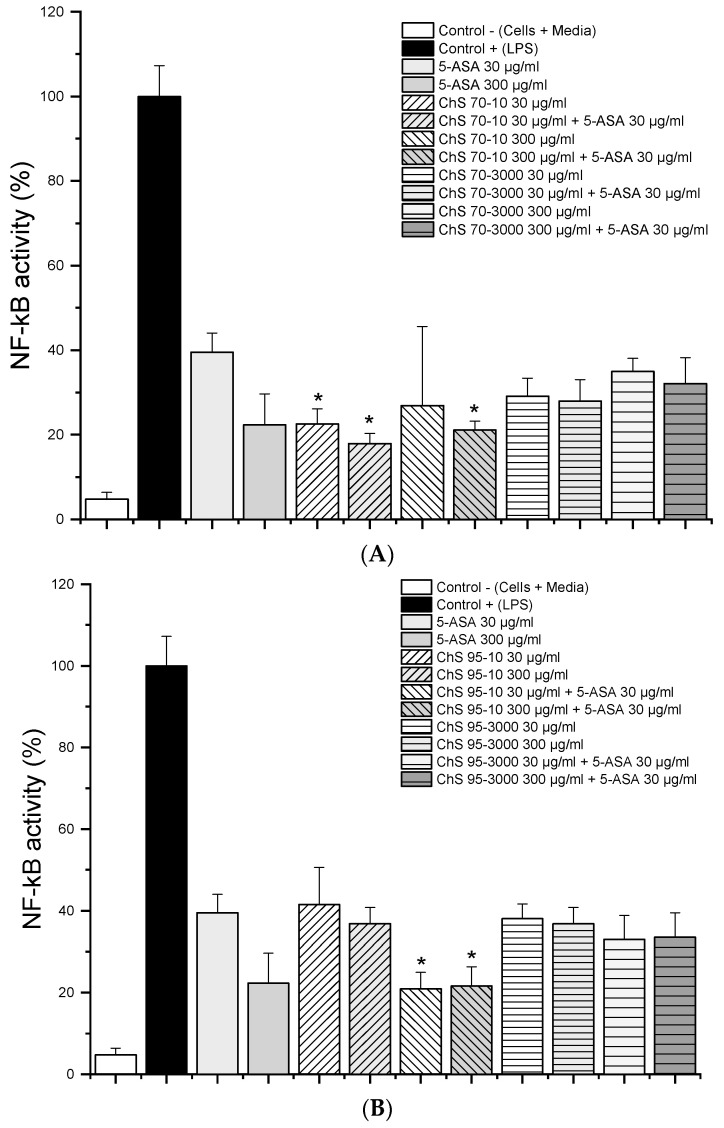
Nuclear factor kappa-B (NF-κB) activity from LPS-stimulated RAW 264.7 macrophages (1 µg/mL) after 24h treatment with 30 µg/mL and 300 µg/mL of chitosan 70% (**A**) and 95% (**B**) DD and viscosity 10 and 3000 mPas and in combination with 30 µg/mL 5-ASA (*n* = 6; data given as mean ± S.D., * = *p* < 0.05 compared to 5-ASA 30 μg/mL alone).

The activity of the transcription factor NF-κB in chitosan grades with 70% DD and 95% DD with a viscosity of 10 and 3000 mPas showed that both grades of chitosan significantly reduced NF-κB levels (Figure 3A,B). The increase in chitosan concentration did not lead to significant effects (except with ChS-95-10) and the addition of 5-ASA did not modify the NF-κB activity dramatically. However, slightly lower NF-κB levels were observed with low-viscosity chitosan grades, while the degree of deacetylation had no significant impact, as observed with 95% DD grades. This clearly differed from observations in TNF-α levels.

### 3.2. Pharmacological Activity In Vivo

The dose of chitosan of 30 mg/kg of all tested grades showed higher survival rates compared to the colitis group that received saline solution (Supplementary Figure S2). Besides, most of the groups receiving chitosan alone fared better than the groups treated with 5-ASA alone, except ChS-70-10, where the survival dropped to 80% on day 5. The groups that received combinations of chitosan with 5-ASA showed no mortality throughout the study period. However, Kaplan–Meier estimates using the log-rank test revealed that the null hypothesis that there are no significant differences between the control colitis groups and the groups treated with 5-ASA and chitosan grades and 5-ASA could not be rejected.

Similarly, treatment with chitosan alone and in combination with 5-ASA was found to lower the clinical activity score (CAS) compared to the untreated control group that received saline solution after induction of colitis (Supplementary Figure S3). The CAS for 5-ASA and the untreated colitis group were 1.8 and 1.9, respectively, on day 5, whereas treatment with chitosan alone or in combination with 5-ASA irrespective of the grade used lowered the CAS to a range of 0.6 to 1.2. Differences were, however, not statistically significant due to very high data variability in each group.

**Figure 4:**
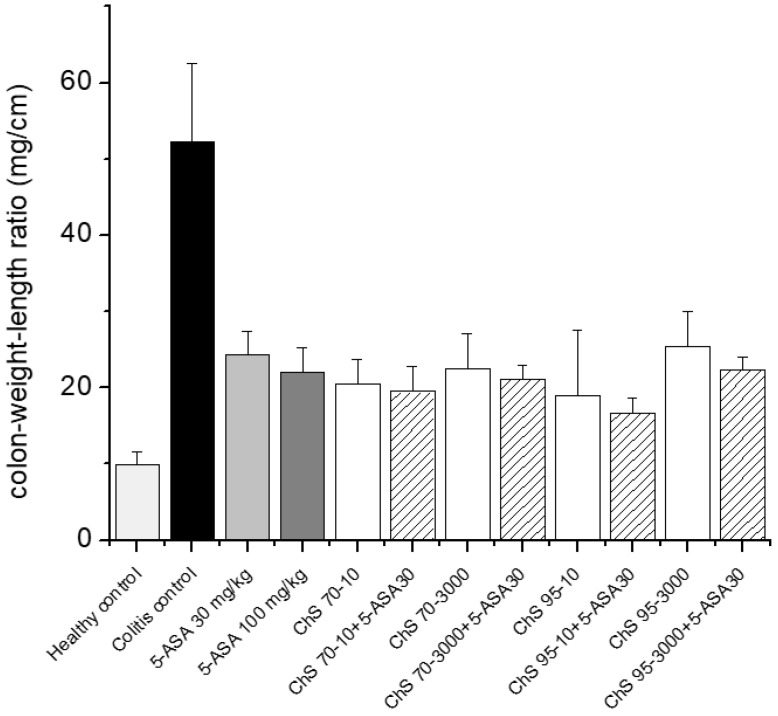
Determination of colon weight length ratio after treatment of experimental colitis (90 mg/kg 2,4,6-trinitrobenzenesulfonic acid (TNBS)) with 30 mg/kg chitosans of different DD (70% and 95%) and viscosities (10 and 3000 mPas) in combination with 30 mg/kg 5-ASA (mean ± SD; *n* = 5; chitosan receiving groups did not differ significantly from 5-ASA 30 mg/kg, Dunnett’s test).

The CWL index was significantly reduced for all chitosan and 5-ASA-treated groups. However, no major differences were observed between the various groups (Figure 4). Similarly, the group receiving 5-ASA alone showed a slightly higher CWL index than chitosan and chitosan + 5-ASA groups, except for ChS-95-3000. Nevertheless, these differences in CWL index were not statistically significant.

Similarly, the appearance of necrosis in macroscopic histological tissue analyses was less prevalent within the treatment groups (II-X) compared to the untreated colitis control group. A distinct visual difference could be observed between treatment with 5-ASA alone (II) compared to chitosan + 5-ASA combinations which exhibited minimal swelling of the colon (Supplementary Figure S4).

**Figure 5:**
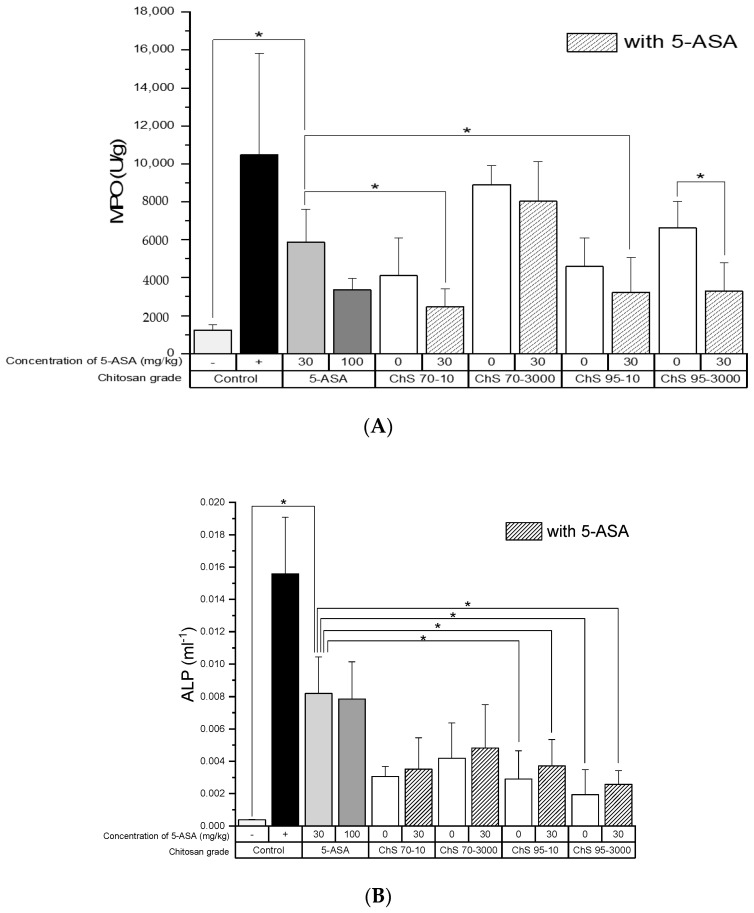
Myeloperoxidase (**A**) and alkaline phosphatase (**B**) activity after treatment of experimental colitis (90 mg/kg TNBS) with chitosans of different DD (70% and 95%) and viscosities (10 and 3000 mPas) at a dose of 30 mg/kg in combination with 5-ASA at a dose of 30 mg/kg, (−): healthy control, (+): untreated colitis control (mean ± SD; *n* = 5; * *p* < 0.05, Dunnett’s test.).

MPO activity was reduced distinctly in all treatment groups compared to the untreated colitis control (Figure 5A) and three of four chitosan and 5-ASA combinations even led to a significant reduction compared to 5-ASA alone. It is worth noting that within the chitosan alone groups, chitosans with lower viscosity or MW showed a better anti-inflammatory effect, but this was not statistically significant.

Generally, ALP showed a comparable trend, i.e., levels in the treatment groups were significantly reduced compared to the untreated colitis control, except for the 5-ASA alone group (Figure 5B). Similarly, compared to the 5-ASA control group, the observed trend was a significant reduction in ALP levels in the groups treated with ChS-95-3000 and ChS-95-3000 + 5-ASA, respectively, as well as for ChS-70-10 + 5-ASA, while all other groups had lowered ALP levels, but not significantly.

**Figure 6:**
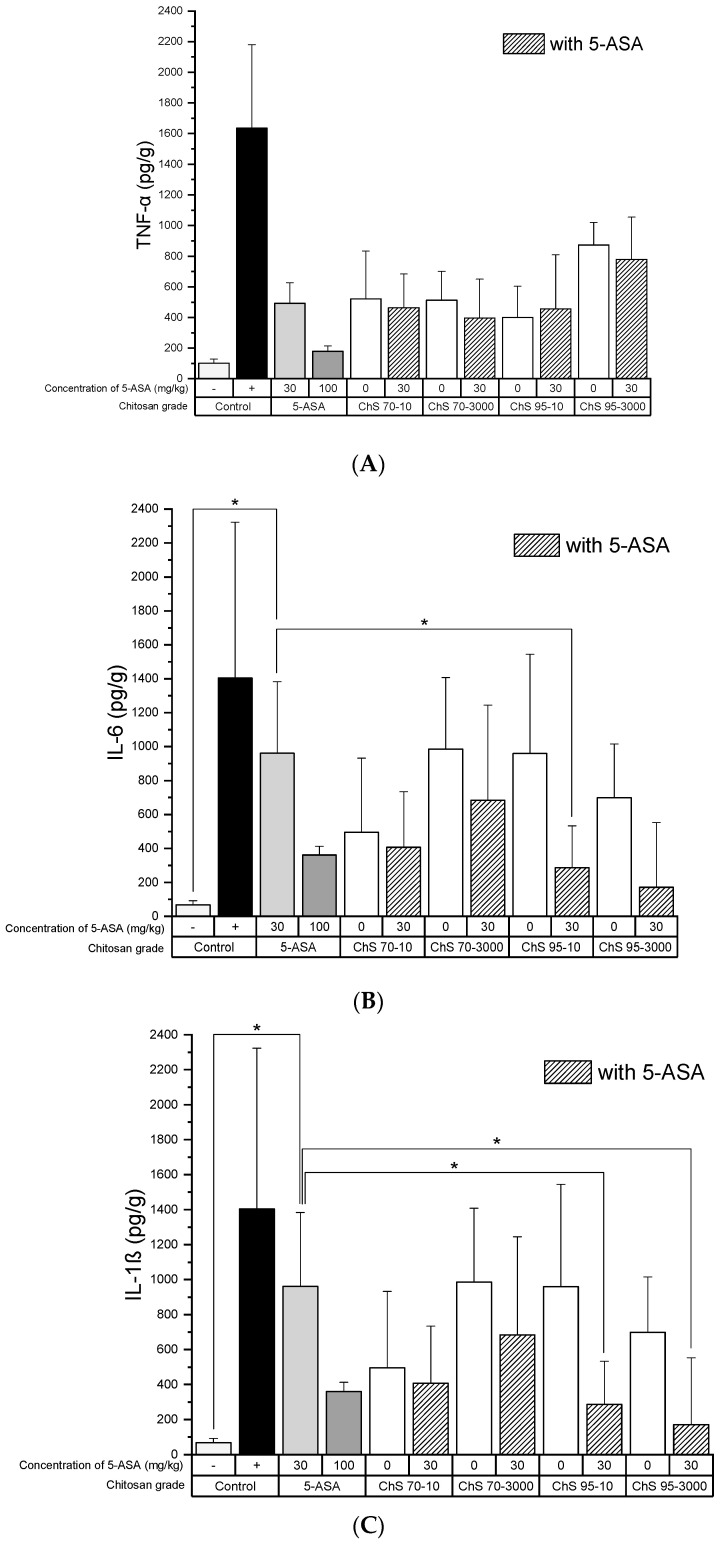
(**A**) TNF-α, (**B**) interleukin-6 (IL-6), (**C**) IL-1β activity in colon tissue homogenates after treatment of experimental colitis (90 mg/kg TNBS) with 30 mg/kg chitosans of different DD (70% and 95%) and viscosities (10 and 3000 mPas) in combination with 30 mg/kg 5-ASA, (−): healthy control, (+): untreated colitis control (mean ± SD; *n* = 5; * *p* < 0.05; Dunnett’s test.).

**Figure 7:**
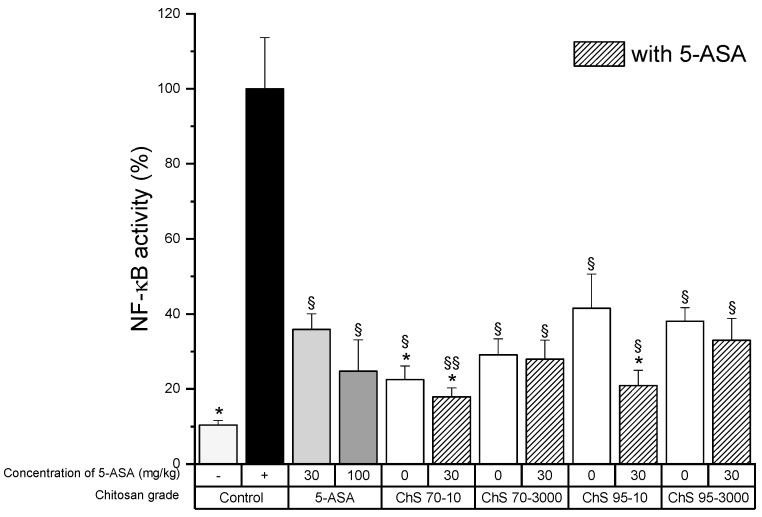
NF-κB activity in colon tissue homogenates after treatment of experimental colitis (90 mg/kg TNBS) with 30 mg/kg chitosans of different DD (70% and 95%) and viscosities (10 and 3000 mPas) in combination with 30 mg/kg 5-ASA, (−): healthy control, (+): untreated colitis control (mean ± SD; *n* = 5; * *p* < 0.05 compared to 5-ASA 30 mg/kg, § *p* < 0.05; §§ *p* < 0.01 compared to colitis control, Dunnett’s test.).

The decrease in the TNF-α levels as well as NF-κB activity was chitosan-grade independent and also the presence of 5-ASA did not alter the outcome compared to the group treated with 5-ASA (Figure 6A and Figure 7). IL-6 levels after treatment with chitosan showed a certain variability, although these were not significantly different from those of 5-ASA alone (Figure 6B).

### 3.3. Bioadhesion Studies

**Figure 8:**
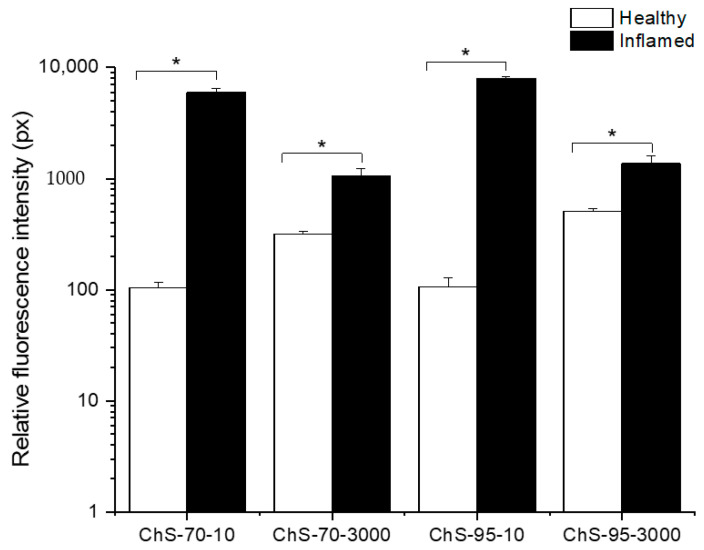
Fluorescence intensity in inflamed tissue for chitosans ChS-70-10, ChS-70-3000, ChS-95-10 and ChS-95-3000 (mean ± SD; *n* = 3; * *p* < 0.05; healthy tissue compared to inflamed tissue for each chitosan).

Qualitative CLSM data suggest that chitosan adheres only to the surface of the epithelium and hardly penetrates into the inflamed regions of the colon (Supplementary Figure S5). In semi-quantitative characterization on colonic bioadhesion, the different chitosans accumulated significantly less in healthy tissues than observed in the colitis groups (Figure 8). Low-viscosity grades showed a higher selectivity (inflamed vs. healthy) compared to high-viscosity grade chitosans, independently from their degree of deacetylation.

## 4. Discussion

The properties of chitosan make it an attractive polymer for biomedical, gene delivery and tissue engineering applications where it has previously been considered as an inactive ingredient.

A few studies have been published on the anti-inflammatory properties of chitosan or its oligosaccharides in IBD [8,11,17], showing that chitosan or its oligosaccharides demonstrate therapeutic efficacy in experimental colitis models. Since no systematic study has been conducted to date to investigate the effect of MW and DD of chitosan in IBD, our study helps to get a better insight into the impact of these molecular properties. Some “stand-alone” studies showed previously that pre-treatment with 20 mg/kg chitosan with a DD of 85–90% resulted in a significant improvement in survival rate, whereas a higher dose of 100 mg/kg was found to be less effective [14]. A similar alleviation of inflammation was observed after chitosan with a DD of 85–90% was administered to mice in DSS-induced colitis [18]. Similarly, a study in acetic acid-induced and DSS-induced colitis showed that oral treatment with low-MW chitosan with a DD of 85–90% at a dose of 20 mg/kg for 5 and 7 days, respectively, led to a significant reduction in MPO activity and a marked improvement in clinical colitis symptoms [8].

The level of MPO and ALP concentrations confirmed that chitosan has its own anti-inflammatory effect in experimental colitis, which is roughly comparable to the effect observed with 5-ASA. Significant differences in these inflammation markers were noted between the combination groups and the group treated with 5-ASA alone. Surprisingly, such an additive effect was not observed as clearly in cytokine levels when chitosan was combined with 5-ASA. Previous studies showed that NF-κB-regulated pro-inflammatory cytokines such as TNF-α in colonic tissues were restored to an almost normal level after treatment with chitosan and confirmed that the suppression of NF-κB activation is likely to be the main signaling pathway that contributes to the protective effect of chitosan in experimental colitis [8,17,19]. This is in line with our findings, although there were no significant differences in the NF-κB activity among the different types of chitosan used, a trend that was also similarly found in cultured macrophages. Furthermore, chitosan is known to act as an immunomodulatory agent by altering the polarized state of macrophages, leading to the release of anti-inflammatory cytokines depending on the prior state of activation of the macrophages [20,21,22]. The ability of chitosan to influence the polarization of macrophages from their M1 to M2 state and to induce a type 1 IFN response leading to the release of anti-inflammatory mediators such as IL-1ra and IL-10 has been previously reported as the mechanism via which chitosan acts as an anti-inflammatory polymer. A downregulation of the TNF-α levels may also be related to an M1 to M2 change.

One noteworthy difference between the TNF-α and NF-κB readout in cell culture is the sensitivity of cells to 95% DD chitosan. A possible explanation is a potential molecular interaction which does not occur with NF-κB, although they are involved in the same pathway. This would also explain the absence of this effect in vivo where the electrostatic interactions of chitosan would be surely hampered by the presence of mucin.

The possible degradation of chitosan by colonic bacterial flora in the inflamed in vivo milieu to its monomeric component glucosamine may lead to the anti-inflammatory effect observed. Glucosamine is involved in tissue repair and wound healing as it not only acts as a substrate for the synthesis of glycosaminoglycans, the building blocks of cell membranes, but also stimulates their synthesis and prevents their degradation, thus contributing to the maintenance of the strength, flexibility and elasticity of tissues [17,23,24,25]. As the higher MW chitosans would require longer to degrade, the kinetics of this degradation reaction would have to be investigated in the inflamed in vivo environment to determine the effect of the treatment time of 3 days on the production of glucosamine. It has been reported that chitosan undergoes microbial degradation in the colon by enzymes such as chitinases and chitosanases [26,27,28,29,30,31] although it is not susceptible to degradation by enzymes in the small intestine [32,33]. It was found that chitosan with a high DD is more easily degraded by chitinases present in the human colon compared to those with low DD [34,35,36]. Therefore, the degradation of chitosan 95-10 into glucosamine and *N*-acetyl glucosamine is likely to be more rapid than that of chitosan 70-10. Glucosamine is known to have anti-inflammatory properties in IBD and in other inflammatory conditions such as arthritis [17,23]. The wound-healing properties of chitosan have been observed in cells with chitosans with a MW of 50–190 kDa and 310–375 kDa. In an in vivo colonic environment, the wound-healing properties of chitosan can be attributed to the fact that chitosan is degraded by the enzymatic action of lysozyme and its monomeric component *N*-acetyl-β-d-glucosamine is further degraded into chitooligomers, which are capable of increasing vascularization, stimulating the correct deposition, assembling and orientating collagen fibrils and being incorporated into the components of the extracellular matrix and reorganizing the tissue architecture [23]. As the higher MW chitosans would require longer to degrade, one would expect that this translates into a visible therapeutic difference. Similarly, it was found that chitosans with a high DD are more easily degraded by chitinases present in the human colon compared to those with low DD [34,35,36]. Therefore, the degradation of chitosan 95-10 into glucosamine and *N*-acetyl glucosamine is likely to be more rapid than that of chitosan 70-10. The absence of any visible impact of the chitosan grades on the therapeutic outcome is not clear. It currently cannot be excluded that two mechanisms are superposed, e.g., active signaling leading to a significant effect for low-viscosity chitosans and a mechanical protective effect in the inflamed tissue could be responsible for the efficacy for the high-viscosity grades. These effects may, however, be subject to change due to the activity of intestinal microflora, which in turn can be further influenced by a variety of parameters, such as nutrition or intake of probiotic food supplements [37].

In experimental IBD, chitosan has been found to suppress the inflammatory response when administered locally to the site of inflammation and, in comparison to 5-ASA, to provide a beneficial limited additive anti-inflammatory effect. A combination of chitosan and 5-ASA in the therapy of IBD could facilitate a dose reduction of 5-ASA, which has been associated with systemic side effects after prolonged use.

## 5. Conclusions

The systematic study showed that the MW and DD have a surprisingly low impact on the anti-inflammatory activity of chitosan in colitis, although low MW (20–100 kDa) chitosan and high DD (95%) showed a certain limited tendency towards better anti-inflammatory activity in IBD with the benefit mainly resulting from combining chitosan with 5-ASA. This shows that this dual combination can offer an additional therapeutic benefit in the treatment of IBD.

## Supplementary Materials

The following are available online at https://www.mdpi.com/1999-4923/12/11/1038/s1, Figure S1: TNF-α expression from LPS-stimulated RAW 264.7 macrophages (1 µg/ml) after 24-hour treatment with increasing concentrations of 5-ASA (*n* = 6; data given as mean ± S.D., * = *p* < 0.05 compared to the LPS control without 5-ASA), Figure S2: Survival rate of mice over experimental period (5 days) after treatment of colitis (90 mg/kg TNBS) with 30 mg/kg chitosans with DD 70% and 95% and viscosity 10 mPas and 3000 mPas respectively and in combination with 30 mg/kg 5-ASA. In all cases treatment with chitosans alone and in combination with 5-ASA led to a 100% survival rate till day 5 except in the case of chitosan-70-10, where the survival rate dropped to 80%. The untreated colitis control group and the 5-ASA group had a survival rate of 60% and 80% respectively. (*n* = 5; * *p* < 0.05, compared to untreated colitis group, log rank test), Figure S3: Clinical activity score during experimental period (5 days) determined for *n* = 5 animals after treatment of experimental colitis (90 mg/kg TNBS) with chitosan grades (ChS-70-10, ChS-70-3000, ChS-95-10, ChS-95-3000) at a dose of 30 mg/kg in combination with 5-ASA at a dose of 30 mg/kg (*n* = 5). Colitis was induced on day 1 using TNBS and treatment was administered on day 2, 3 and 4. Animals were sacrificed on day 5. Error bars were omitted for clarity reasons., Figure S4: Photographs representative of the mouse colon show tissue sections from the (I) control (untreated) colitis group, (II) group treated with 30 mg/kg 5-ASA, (III) 30 mg/kg chitosan 70-10, (IV) 30 mg/kg chitosan 70-10 and 30 mg/kg 5-ASA, (V) 30 mg/kg chitosan 70-3000, (VI) 30 mg/kg chitosan 70-3000 and 30 mg/kg 5-ASA, (VII) 30 mg/kg chitosan 95-10, (VIII) 30 mg/kg chitosan 95-10 and 30 mg/kg 5-ASA, (IX) 30 mg/kg chitosan 95-3000 and (X) 30 mg/kg chitosan 95-3000 and 30 mg/kg 5-ASA in experimental colitis induced using 90 mg/kg TNBS, Figure S5: CLSM images obtained after intrarectal administration of labelled chitosan stained with FITC and shown in green to healthy mice and TNBS-induced colitis mice. Images A and B were obtained after administration of chitosan 70% DD and 10 mPas to healthy and colitis mice. Similarly, images C and D were obtained after administration of chitosan 70% DD and 3000 mPas, E and F were obtained after administration of chitosan 95% DD and 10 mPas and G and H were obtained after administration of chitosan 95% DD and 3000 mPas to healthy and colitis mice, Table S1: Physicochemical properties of chitosan grades.
