# Supplementary material for: Anti-Inflammatory Activity of Chitosan and 5-Amino Salicylic Acid Combinations in Experimental Colitis
Source: Pharmaceutics. 2020 Oct 29;12(11):1038. doi: 10.3390/pharmaceutics12111038 (PMC7692366; doi:10.3390/pharmaceutics12111038)
Supplement: Supplementary file 1 [file pharmaceutics-12-01038-s001.pdf]

pharmaceutics

MDPI

Supplementary Materials: Anti-Inflammatory Activity of Chitosan and 5-Amino Salicylic Acid Combinations in Experimental Colitis

Henusha D. Jhundoo, Tobias Siefen, Alfred Liang, Christoph Schmidt, John Lokhnauth, Arnaud Béduneau, Yann Pellequer, Crilles Casper Larsen and Alf Lamprecht

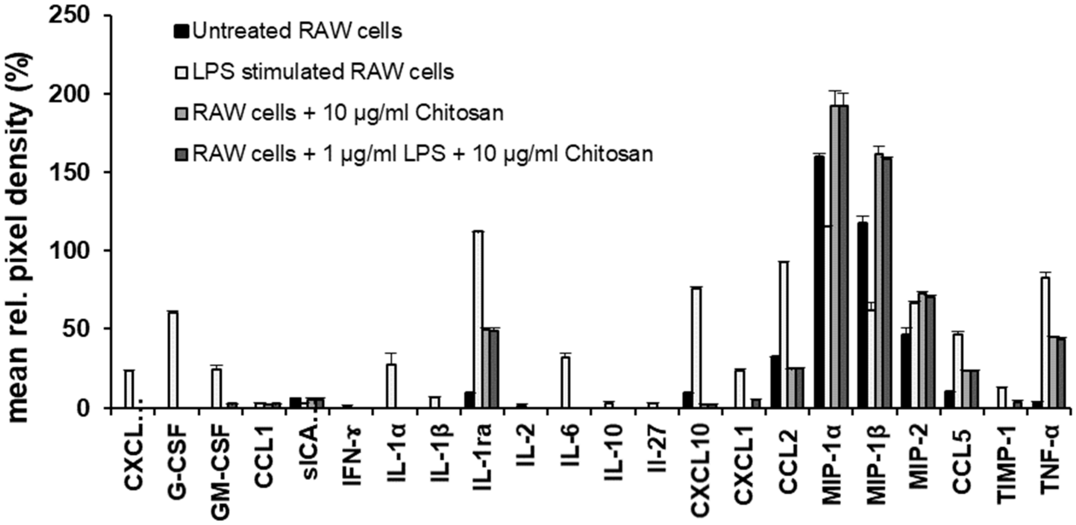

Figure S1. Cytokine array for mouse RAW 264.7 macrophage cells that were either untreated, treated with 1 µg/ml LPS for 24 h, treated with 10 µg/ml chitosan for 24 h or treated with 1 µg/ml LPS for 24 h and with 10 µg/ml chitosan for another 24 h.

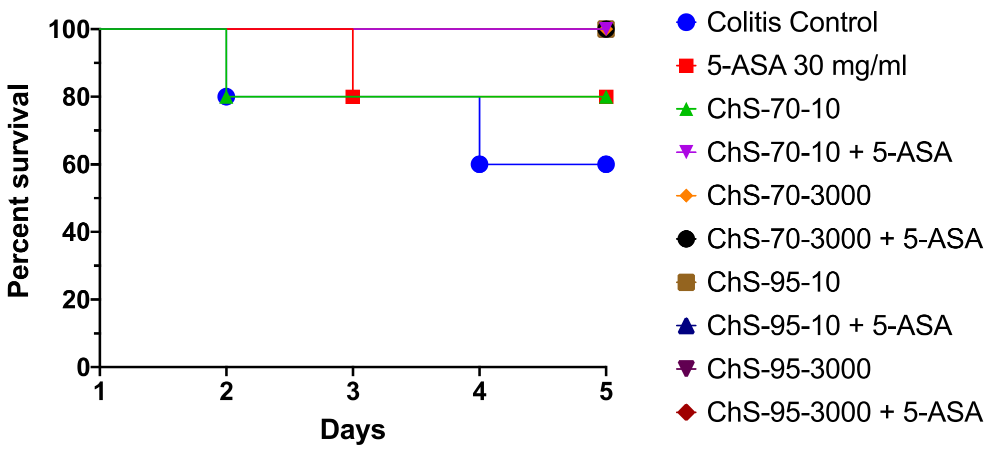

Figure S2. Survival rate of mice over experimental period (5 days) after treatment of colitis (90 mg/kg TNBS) with 30 mg/kg chitosans with DD 70% and 95% and viscosity 10 mPas and 3000 mPas respectively and in combination with 30 mg/kg 5-ASA. In all cases treatment with chitosans alone and in combination with 5-ASA led to a 100% survival rate till day 5 except in the case of chitosan-70-10, where the survival rate dropped to 80%. The untreated colitis control group and the 5-ASA group had a survival rate of 60% and 80% respectively. ($n = 5$; * $p < 0.05$, compared to untreated colitis group, log rank test).

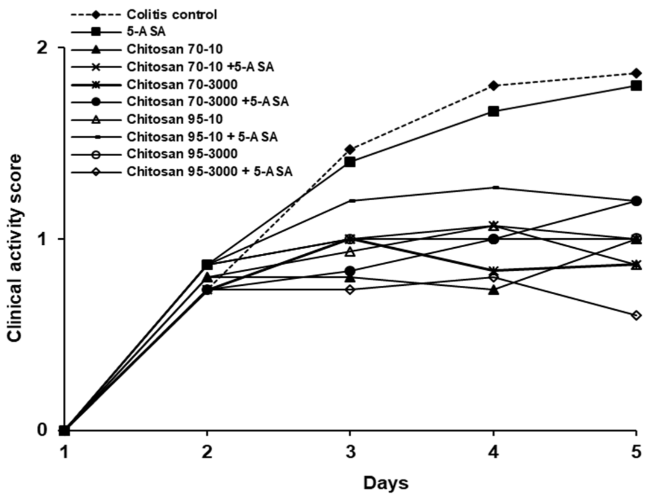

Figure S3. Clinical activity score during experimental period (5 days) determined for $n = 5$ animals after treatment of experimental colitis (90 mg/kg TNBS) with chitosan grades (Ch-70-10, Ch-70-3000, Ch-95-10, Ch-95-3000) at a dose of 30 mg/kg in combination with 5-ASA at a dose of 30 mg/kg ($n = 5$).Colitis was induced on day 1 using TNBS and treatment was administered on day 2, 3 and 4. Animals were sacrificed on day 5. Error bars were omitted for clarity reasons.

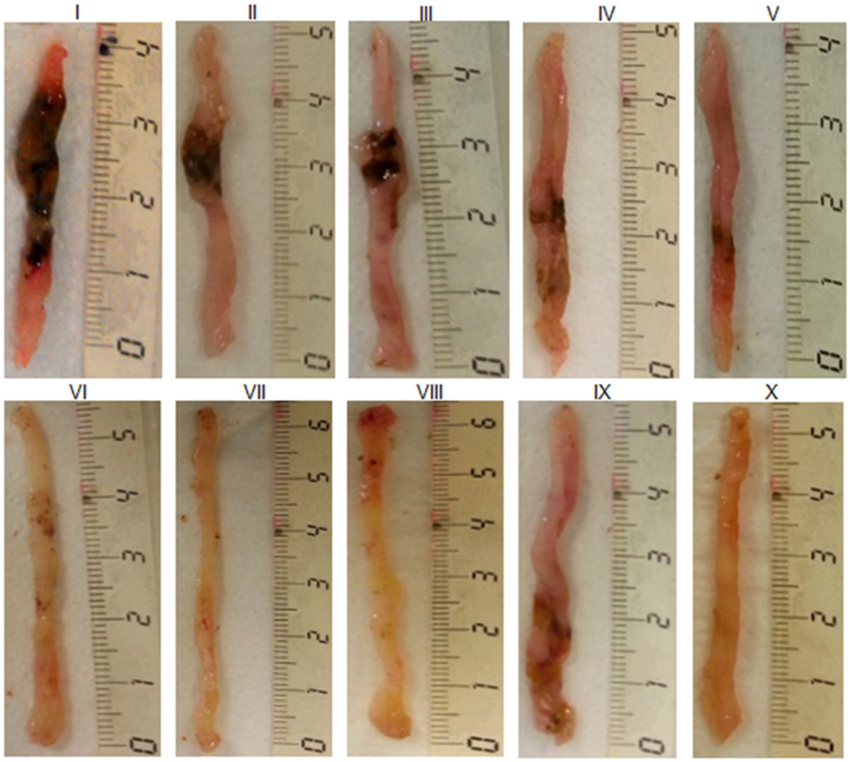

Figure S4. Photographs representative of the mouse colon show tissue sections from the (I) control (untreated) colitis group, (II) group treated with 30 mg/kg 5-ASA, (III) 30 mg/kg chitosan 70-10, (IV) 30 mg/kg chitosan 70-10 and 30 mg/kg 5-ASA, (V) 30 mg/kg chitosan 70-3000, (VI) 30 mg/kg chitosan 70-3000 and 30 mg/kg 5-ASA, (VII) 30 mg/kg chitosan 95-10, (VIII) 30 mg/kg chitosan 95-10 and 30 mg/kg 5-ASA, (IX) 30 mg/kg chitosan 95-3000 and (X) 30 mg/kg chitosan 95-3000 and 30 mg/kg 5-ASA in experimental colitis induced using 90 mg/kg TNBS.

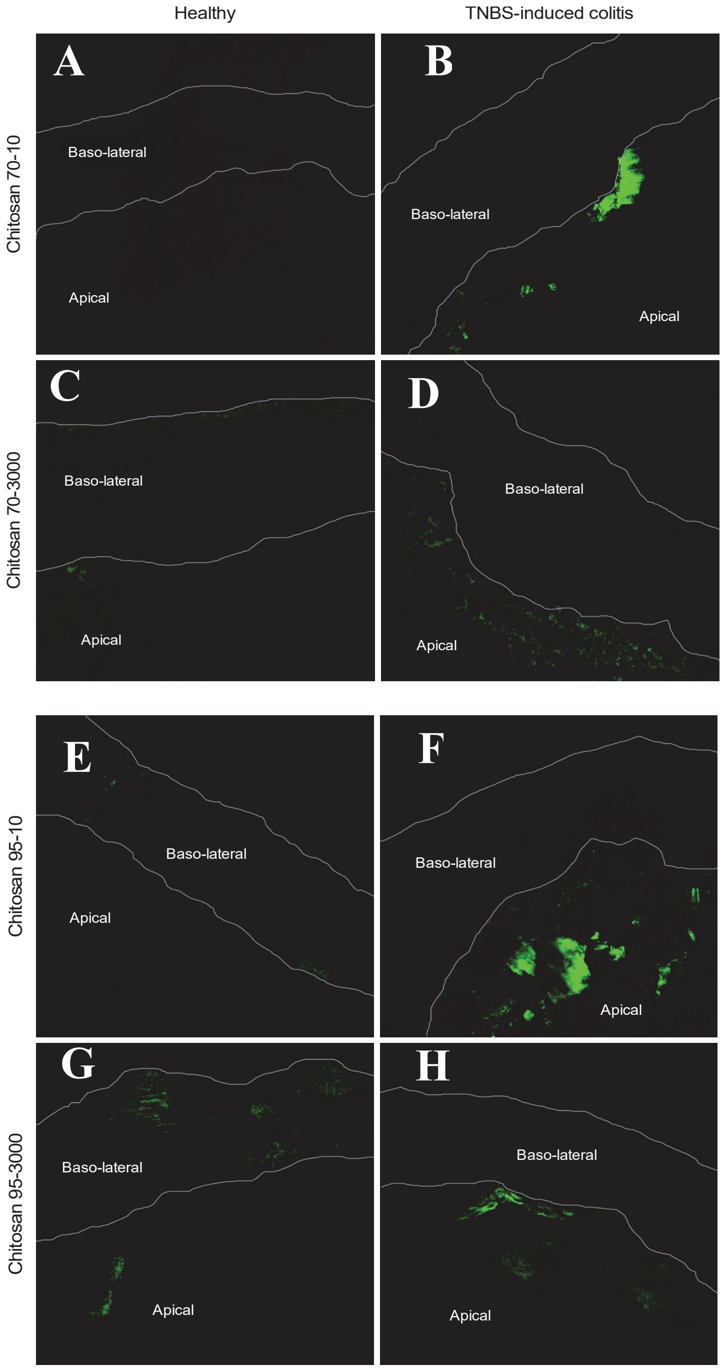

Figure S5. CLSM images obtained after intrarectal administration of labelled chitosan stained with FITC and shown in green to healthy mice and TNBS-induced colitis mice. Images A and B were obtained after administration of chitosan 70% DD and 10 mPas to healthy and colitis mice. Similarly, images C and D were obtained after administration of chitosan 70% DD and 3000 mPas, E and F were obtained after administration of chitosan 95% DD and 10 mPas and G and H were obtained after administration of chitosan 95% DD and 3000 mPas to healthy and colitis mice.

Table S1. Physicochemical properties of chitosan grades.

**Chitosan (ChS)**	**Molecular weight (M_w by GPC, kDa)**	**Viscosity (1% *w/v* in 1% *v/v* acetic acid, mPas)**	**Degree of deacetylation (%)**
Chitosan-70-10	20-100	8-15	≤ 67.6-72.5
Chitosan-70-500	200-400	351-750	≤ 67.6-72.5
Chitosan-70-3000	250-550	2751-3250	≤ 67.6-72.5
Chitosan-95-10	20-100	8-15	≥ 92.6
Chitosan-95-500	200-400	351-750	≥ 92.6
Chitosan-95-3000	250-550	2751-3250	≥ 92.6
